# The sound of yawns makes geladas yawn

**DOI:** 10.1038/s41598-023-49797-5

**Published:** 2024-01-07

**Authors:** Luca Pedruzzi, Martina Francesconi, Elisabetta Palagi, Alban Lemasson

**Affiliations:** 1 Unit of Ethology, Department of Biology, University of Pisa, 56126 Pisa, Italy; 2 CNRS, EthoS (Ethologie Animale et Humaine)-U.M.R 6552, Université de Rennes, Université de Normandie, 35000 Rennes, France; 3 Natural History Museum, University of Pisa, 56017 Calci, Pisa, Italy; 4 Institut Universitaire de France, 1 Rue Descartes, 75231 Paris Cedex 05, France

**Keywords:** Social evolution, Social behaviour, Zoology, Animal behaviour

## Abstract

Yawning is undeniably contagious and hard to resist. Interestingly, in our species, even the mere sound of a yawn can trigger this contagious response, especially when the yawner is someone familiar. Together with humans, one other mammal species is known to produce loud and distinct vocalisations while yawning, *Theropithecus gelada.* Geladas are known for their complex social interactions and rich vocal communication, making them intriguing subjects for studying yawning behaviour. To explore the contagious effect of yawn sounds on geladas, we conducted playback experiments in a zoo-housed colony with animals living in two groups. We exposed them to yawn sounds (Test) or affiliative grunts (Control) produced by males from either their own group or the other one. The results were remarkable, as simply hearing yawn sounds led to yawn contagion in geladas, with multiple responses observed when the yawns came from members of their own group. This finding adds a significant contribution to the research on mimicry and behavioural contagion in primates. Moreover, it raises intriguing questions about the involvement of sensory modalities beyond visual perception in these phenomena.

## Introduction

Yawning makes you yawn, we have all experienced that. The phenomenon of yawn contagion (YC) is not restricted to humans and seem present mostly in highly social species, across the primate lineage (great apes^1^, cercopithecines^2–4^, recently in a south American monkey^5^ and in a lemur species^6^) as well as in species from other orders (e.g., pigs^7^, wolves^8^, domestic dogs^9^, lions,^10^, spotted hyenas^11^, African painted dogs^12^, budgerigars^13^). Despite the mysteries around the possible functions and neurobiology underpinning YC, recent findings suggest that the phenomenon could have evolved to promote group synchronization^10^ and that it correlates to a certain extent with social closeness^14^. For humans, the phenomenon seems exaggerated as even just hearing someone yawning can elicit YC, with this getting easier when the yawners are familiar^15,16^.

**Figure 1:**
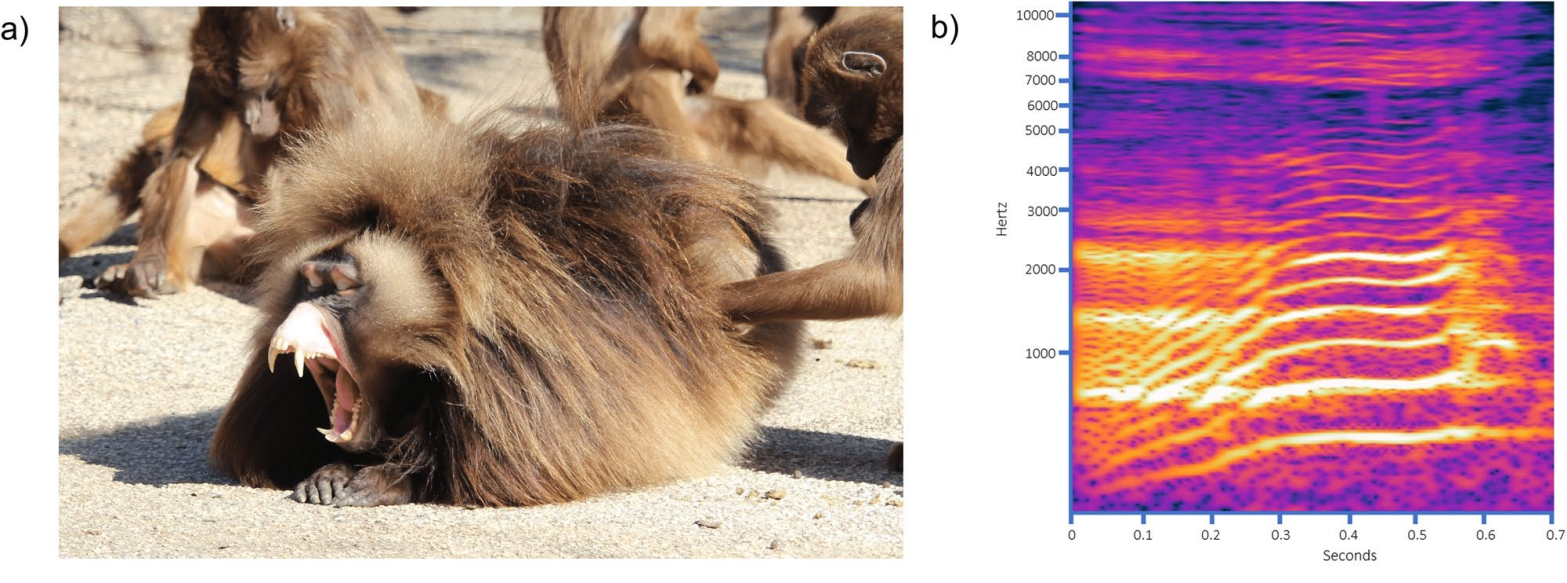
(**a**) Male gelada yawning (NaturZoo Rheine, credits: M. Francesconi). (**b**) Spectrogram of a yawn vocalisation produced by a gelada male.

Vocalized yawns are not restricted to humans; indeed, another mammal species, *Theropithecus gelada*, also emits evident vocalizations while yawning^17^. The gelada (Fig. 1a) is an Ethiopian endemic monkey species living in multi-level societies (e.g., units, teams, bands, communities). The core unit of gelada groups can be either the one-male unit, composed of a reproductive adult male, adult females, their offspring, and eventually one or more follower males, or the all-male unit, where subadult or young adult males gather after dispersion from the natal unit^18^. Moreover, the species show fission–fusion dynamics between the different one-male and all-male group units^18^. Among primates, geladas are particularly well known for their social complexity and rich vocal communication^19^. Indeed, when compared to phylogenetically close taxa (e.g., *Papio* and *Lophocebus* genera,^20^), they are characterized by a relatively richer vocal repertoire, including their own derived sound types^17^, and they have often been compared to our species, not only for their social^18^, but also for their communicative complexity^19^. A similar evolutionary social landscape, with similar challenges (e.g., need of group coordination with subjects not always in visual contact), has indeed possibly led to the emergence of multimodal communication in both species. In geladas, the acoustic component of yawns is not a mere by-product of the inhalation/exhalation cycle but it represents a distinct vocalisation which is mainly produced by males (File S1, Fig. 1b)^17^. Vocalised yawns represent a conspicuous signal^21,22^ often audible at notable distances for their loudness (e.g., > 40 m,^2^), further hinting at a possible role of yawning in communication and coordination. In this framework, these peculiar vocalisations could thus be a social outcome of the convergent evolution experienced by humans and geladas, both characterised by derived acoustic repertoires^17,23^. Although we know that geladas yawn after seeing others’ yawns^2,3^, we do not know if the sole acoustic component can elicit contagion, as little is known about the role that different sensory modalities play in yawn contagion and other mimicry phenomena^24^. Among non-human animals, domestic dogs are known to be susceptible to human yawn sounds^9,25,26^. Yet, intraspecific auditory YC remains unexplored in non-human animals. The unique trait of yawn vocalisation and the analogies with humans suggest that the vocalisation might have evolved to make contagion possible also when the subjects are not in visual contact and thus, we expected that the sole auditory component could induce YC. Moreover, as it occurs with human yawn sounds (human–human YC^16^, human–dog YC^9,26^), we could expect auditory YC to be modulated according to the degree of social value of the trigger. Here, to test such hypotheses we carried out playback experiments to verify the presence of acoustic-based yawn contagion in a zoo-housed colony of geladas, with the animals belonging to two groups housed in adjacent open enclosures without visual and acoustic barriers. Specifically, in separate randomized sessions we exposed the animals to the sound of yawns (Test) *vs* affiliative grunts (Control) produced by in- or outgroup males.

## Methods

### Study subjects and housing conditions

The present study was carried out in April–May 2023 at NaturZoo Rheine (Germany), where the world largest colony of geladas is hosted (n = 106 animals)^28^. At the time of data collection, the colony was divided into two enclosures (G1 and G2). G1 was composed of 2 one-male units and G2 was composed of 2 one-male units and 1 all-male unit (Table S1). A pond of water divides the two enclosures, and thus animals of the two groups can hear and see each other but cannot enter in contact. This setting represents a unique opportunity with all the units acoustically familiar to each other but with different levels of spatial association. Each enclosure had both indoor (36 m^2^) and large outdoor spaces (an island of 2700 m^2^ surrounded by a boundary ditch). The animals were free to move in both the indoor and outdoor areas. Stimuli recording and playback sessions were only carried out in the outdoor facility. All the adult subjects of the colony were individually recognized by LP and MF. Among these, 33 randomly chosen subjects of both sexes and belonging to all the group units (7 males and 26 females, 21 subjects from G1, 12 subjects from G2) underwent playback experiments. Since yawn contagion in geladas is only present in adults^3^, we selected subjects from this age class.

### Stimulus preparation and experimental setting

We selected male yawn vocalisations as Test stimuli. We decided to use male stimuli because yawn sounds are mostly produced by males^17^ and can have a role in group coordination^2^. Among gelada vocal repertoire, we chose male affiliative grunts as Control vocalisations since these have a neutral to slightly positive valence, and they are often produced by geladas^17^. Despite their valence possibly being slightly different from that of yawns, we tried to minimize the issue by excluding yawns produced in potentially negative conditions (e.g., being involved or witnessing aggression, see below). To prepare acoustic stimuli, male vocalisations were collected with a directional microphone (Sennheiser MKE600) connected to a handy recorder (ZOOM H5, sample rate: 44,100 Hz, resolution: 16-bit, wav format) during spontaneous interactions (distance from the animals varied from 5 to 15 m).

During the recording phase, we also measured the loudness (decibel, dB) of both yawn vocalisations and grunts (mean ± SD = 54.0 ± 6.6 dB) with a professional sound meter (SLM-25, Gain Express Holdings). Vocalisations produced during or in the 3-min period after aggressions were excluded from the stimulus pool. Only stimuli with a high sound-to-noise ratio were kept (e.g., no birds or vocalisations from other geladas in the background). The recordings were edited using Audacity software (Audacity version 3.3.2), creating a pool of Test and Control stimuli produced by each of the five fully adult males leading the five group units of the colony. The amplitude of all stimuli was normalised so as to reach about 54.0 dB at the tested subject location (distance from the speaker varied from 5 to 15 m).

Each stimulus was composed of three male yawn vocalisations or three grunt pairs. As grunts are shorter than yawn sounds and normally produced in sequence of two or more grunts^17^, to make Test and Control comparable, grunt pairs were chosen. All the vocalisations composing a given stimulus were produced by the same male. Yawn sounds and grunt pairs composing a certain stimulus were separated one another by a silence of five to ten seconds (randomized). For each experimental session (stimulus presentation and recording of subsequent three minutes), both yawns and grunts were randomly chosen from a pool of at least 15 vocalisations produced by each of the five males. A given vocalization was never used twice during the entire experimental session.

Each tested subject could thus undergo four different conditions (yawn/control stimulus from an outgroup male, yawn/control stimulus from an ingroup male), replicated for each of the five adult males in the stimuli. Females (n = 26) and not leader males (n = 2) could thus undergo ten different conditions, whereas fully adult males (n = 5) eight conditions (as subjects were never tested with their own voice).

After the stimulus was broadcasted, we recorded (SONY handy-cam Full HD, FDR-AX43A) the behaviour of the tested subject for the following three minutes, in accordance with the latency of (visual) yawn contagion extending to the first three minutes after stimulus perception^2,28^.

Several precautions were taken to limit confounding factors during playback sessions. Playback sessions were always carried out far from feeding time (at least 30 min before the start of feeding or at least 30 min after almost complete food consumption). Tested subjects were followed for 3 min before starting the playback so that we could be sure they were not already yawning before the session. We opportunistically performed playback experiments when a subject was relatively far from the group and not socially interacting with other animals. The stimulus broadcasted was then randomly chosen among the possible conditions, so that each subject experienced the different conditions in a random order to limit habituation bias.

Several other precautions were adopted to limit confounding factors. We did not perform sessions when aggression took place in the group (at least three minutes before). Sessions were discarded if aggression took place during them, or when the subject was not visible for the entire duration of the session. The variable *distance from others* was measured to indicate whether other subjects were present in the video (> 2 m from the tested subject), close to the tested subject (< 2 m) or if no other subjects were present in the video recording of the session. The first experimenter video-recorded the playback sessions with a similar zoom so as to cover a 5-m radius around the focal tested subject; concurrently, the second experimenter checked whether other non-tested subjects yawned during the session. In case of sessions in which a non-focal subject yawned, these were discarded (n = 28 sessions) if the yawner was visible and/or audible to the tested individual. Throughout the different sessions, the relative position of the two experimenters was always comparable and standardized (i.e., the two opposite sides of the enclosure) and remained so for all the experimental session (stimulus presentation + following three-min time slot).

In total, this led us to 320 experimental sessions on 33 subjects. During playbacks, the speaker (MiPRO MA-100 single channel Personal Wireless PA system) was positioned so that the sound would appear to come from the direction of the subject yawning in the stimulus, to make it reliable and not violating expectation. The speaker was not visible by geladas (hidden in vegetation) and stimuli were produced at distance via Bluetooth.

### Video coding

All the videos were named by EP with labels not corresponding to the condition of the stimulus presented, so that LP, who carried the scoring on muted videos, was completely unaware of the content of the session (blind analysis). All yawning events, and precise time (mm:ss.000) of occurrence were coded. Then, the total amount of time spent by tested subjects in self-directed behaviours (self-grooming, self-scratching, head shakings, proxy indicators for the anxiety state in monkeys^29^) was quantified (seconds). Self-directed behaviours were coded as they can act as a confounding factor in the study of YC^30^. Inter-observer reliability was then measured with MF, who coded in the same conditions of LP, 20% of the videos. Cohen’s coefficient^31^ was 0.948 for yawning events and 0.971 for self-directed behaviours.

### Statistical analyses

Subjects who received less than 4 playback sessions (and that did not receive at least one Control and one Test stimulus from Ingroup and Outgroup males) were excluded (n = 5), leading to n = 28 subjects and n = 310 playback sessions. We ran a Generalized Linear Mixed Model (glmmTMB 1.2.5042 package on R) to evaluate which variables affected the likelihood of yawning during a session (GLMM_yawn response,_ absence/presence of yawning, binomial distribution of the response variable). Then, we ran a Generalized Linear Mixed Model with a Poisson error distribution for zero-inflated data^32^, to evaluate which factors affected the number of yawns produced during the session (GLMM_number of yawns_). Importantly, to avoid pseudo-replication issues, the interaction between the *tested subject identity* (since not all 28 tested subjects underwent the same number of sessions) and *stimulus yawner identity* was included in the models. Moreover, to avoid possible confounding factors, the *order* of the sessions per each subject and the *distance from others* were also included as random factors. For both GLMM_yawn response_ and GLMM_number of yawns_, the fixed factors were (1) the three-way interaction between the *Sex* of the tested subject (Male, Female), the *Condition* of the stimulus (Control, Test), and the *Group* membership of the trigger stimulus (Ingroup, Outgroup), (2) the time spent in self-directed behaviours in the 3-min after stimulus presentation (*Sdb,* seconds), and (3) the time of the day of the session (hours). We checked for multicollinearity in the GLMMs not including the interaction terms^33^ using the ‘check_collinearity’ function (R package performance 0.4.4). ‘Low correlation’ was found for all the fixed factors in the two models (VIF range: 1.01–1.08). We tested the models’ significance by comparing the full with the control model (i.e., only including random factors and the offset)^34^ through the Likelihood Ratio Test (LRT, Anova with the ‘Chisq’ argument^35^). We estimated the p-values of each predictor and of all interactions running LRTs between the full model and the model not containing that predictor^36^. Then, we also calculated (R-package MuMIn 1.43.17^37^) the marginal R^2^ (proportion of variance of the response variable explained by the fixed factors only) and residual R^2^ (variance of response variable explained by both fixed and random factors)^38^. In GLMM_yawn response_, relative odds ratios were used to demonstrate the influence of fixed factors through the *confint()* function. Odds ratios (OR) depict the anticipated shift in odds when all variables are maintained at reference values, and the fixed factor undergoes a categorical level change.

### Ethical statement

Despite the study being completely non-invasive, a formal approval was asked and received by the Bioethical committee of the University of Pisa (OPBA, n. 14/2023).

## Results

### GLMM_yawn response_

**Figure 2:**
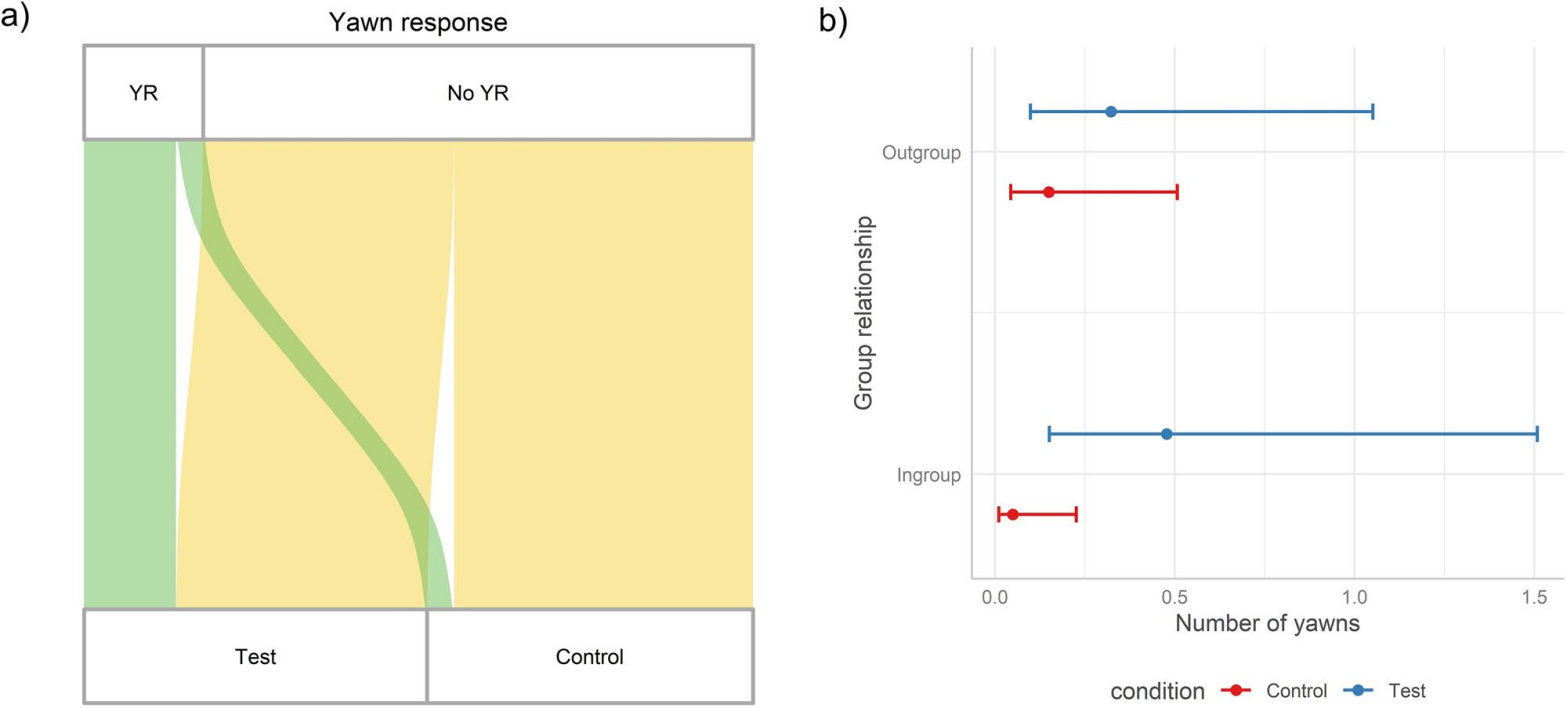
(**a**) Alluvial plot showing the occurrence of yawn response (YR = sessions with yawn responses, No YR = sessions without yawn responses) for each level of the factor *Condition*, showing the different occurrence of yawn responses when tested subjects were exposed to Test vs Control stimuli (green streams = proportion of sessions with presence of yawn response by the tested subject; yellow streams = proportion of sessions with absence of yawn response by the tested subject), thus representing the significant effect of *Condition* in GLMM_yawn response_ (*Condition*: X^2^ = 18.54, p < 0.001). (**b**) Effect plot showing the significant interaction between the *Condition* and the *Group* membership of the stimulus yawner (GLMM_number of yawns_, Group*Condition: X^2^ = 4.69, p = 0.03).

The full model investigating what affected the likelihood of yawning response was significantly different from the null one (χ^2^_9_ = 45.52, p < 0.001, R^2^ marginal = 0.239, R^2^ conditional = 0.481). The significant fixed factors were the *Condition* of the stimulus and the *Sex* of the tested subject (Table 1a). Going into specifics, compared to control grunts, yawn sounds increased the likelihood of yawning more than fourfold (odds ratio = 4.29) (Fig. 2a, GLMM_yawn response_, *Condition*: X^2^ = 18.54, p < 0.001). Importantly, self-directed behaviors (*Sdb*) did not affect the yawning response of tested subjects (χ^2^ = 2.55, p = 0.11, see Fig. S1), thus suggesting that the higher probability of yawn responses under Test condition is independent from the affective state of the subject. None of the combinations of interactions was significant (Group*Condition, χ^2^ = 0.21, p = 0.64, Sex*Group, χ^2^ = 0.35, p = 0.55, Sex*Condition, χ^2^ = 1.80, p = 0.18, Sex*Group*Condition, χ^2^ = 0.03, p = 0.87). Males produced more yawn responses than females but did so independently from the *Condition* of the stimulus (i.e., the effect of the condition did not differ between the two sexes). Full results in Table 1a.

**Table 1:** Estimated parameters (estimate), standard error (SE), and results of the likelihood ratio tests (χ^2^) of the GLMMs (Poisson error distribution) with (a) *Yawn response* as response variable (binomial distribution, GLMM_yawn response_, n = 310), and (b) *Number of yawns* as response variable (zero-inflated Poisson distribution, GLMM_number of yawns_, n = 310).

Fixed factors	Estimate	SE	*df*	χ^2^	*p-*value
(a) GLMM _yawn response_. Random factors: tested subject identity (variance = 0.027, SD = 0.164); stimulus yawner identity (var. = 0.301, SD = 0.549); distance from others (var. = 0.882, SD = 0.940); order (var. = 0.330, SD = 0.574)					
Intercept	− 1.986	1.156	–	–	–
Condition^a,b^ (Test)	1.459	0.055	1	18.541	**0.000**
Sex^a,b^ (Male)	0.582	1.033	1	16.657	**0.000**
Time	− 0.095	0.055	1	2.988	0.084
Sdb	− 0.011	0.007	1	2.552	0.110
Group^a,b^ (Outgroup)	− 0.140	0.904	1	0.020	0.890
Condition*Group	− 0.247	1.086	1	0.214	0.644
Condition*Sex	1.214	1.206	1	1.799	0.180
Group*Sex	0.673	1.351	1	0.353	0.553
Condition*Group*Sex	− 0.262	1.589	1	0.027	0.869
(b) GLMM _number of yawns_. Random factors: tested subject identity (variance = 0.131, SD = 0.362); stimulus yawner identity (var. = 0.196, SD = 0.442); distance from others (var. = 0.655, SD = 0.809); order (var. = 0.495, SD = 0.704)					
Intercept	− 2.602	0.932	–	–	–
Condition^a,b^ (Test)	1.322	0.654	1	17.179	**0.000**
Sex^a,b^ (Male)	0.134	0.954	1	27.016	**0.000**
Time	− 0.034	0.034	1	0.986	0.321
Sdb	− 0.003	0.004	1	0.542	0.461
Group^a,b^ (Outgroup)	− 0.082	0.789	1	0.416	0.519
Condition*Group	− 0.605	0.916	1	4.691	**0.030**
Condition*Sex	1.380	1.002	1	0.778	0.378
Group*Sex	1.734	1.122	1	1.482	0.223
Condition*Group*Sex	− 1.306	1.231	1	1.125	0.289

Significant values are in bold.

^a^Estimate ± SE refer to the difference of the response between the reported level of this categorical predictor and the reference category of the same predictor.

^b^These predictors were dummy coded, with the “Condition (Control)”, “Sex (Female)”, “Group (Ingroup)”, being the reference categories.

### GLMM_number of yawns_

The full model investigating what affected the number of yawns produced by the tested subject was significantly different from the null one (χ^2^_9_ = 61.96, p < 0.001, R^2^ marginal = 0.224, R^2^ conditional = 0.545). The significant fixed factors were the *Condition* of the stimulus (χ^2^ = 17.18, p < 0.001) and the *Sex* of the tested subject (χ^2^ = 27.02, p < 0.001). Among the combinations of interactions considered, the Group*Condition was significant (χ^2^ = 4.69, p = 0.03, Fig. 2b), whereas Sex*Group (χ^2^ = 1.48, p = 0.22), Sex*Condition (χ^2^ = 0.78, p = 0.38), Sex*Group*Condition (χ^2^ = 1.12, p = 0.29) were not. Subjects produced a higher number of yawns when exposed to Test than Control stimuli. As in the previous GLMM, male geladas emitted more yawns compared to females and, again, this effect was independent from the *Condition* of the stimulus. Notably, the effect of the *Condition* was stronger when the stimuli were emitted by ingroup compared to outgroup males (Fig. 2b). Full results in Table 1b.

## Discussion

Through a playback experiment, we made a noteworthy discovery: auditory yawn contagion (YC) between conspecifics extends beyond our species. The mere sound of a yawn can trigger contagious yawning in geladas. When compared to control grunts, yawn sounds induced a higher probability and a greater number of yawns in both male and female individuals, with similar contagiousness observed for both the two sexes. Interestingly, we found that neither self-directed behaviours (clues of anxiety states in primates, as described in^29^) nor the time of the day (which generally influences the frequency of spontaneous yawning, as reported in^22^) had an impact on the yawning responses. This is important to consider, as yawning in primates has often been associated with anxious or slightly negative states^39^, potentially leading to non-conclusive evidence of contagion^30^. Thus, we ruled out these possible confounding effects. Regarding the group membership of the stimulus yawner, we observed that it did not affect the likelihood of the yawning response. However, yawn sounds from individuals within the same group elicited a higher number of yawns compared to yawn sounds from individuals outside the group. In agreement with previous findings on social modulation of YC based on visual cues, our data extend the effect of group membership also to YC based on acoustic cues (^14^ but see also^40^).

The reason behind geladas and humans producing specific vocalizations while yawning remains an intriguing puzzle. However, the unique multimodal nature of this trait suggests a social function in both species. The finding that the sole acoustic component can induce yawn contagion (YC) provides additional evidence for the behaviour's potential role in regulating social interactions. Considering the significance of yawning in group synchronization^10^, the adaptive value of auditory YC might be linked to the need for individuals to maintain acoustic contact when visual contact is not possible, making this especially relevant in societies characterized by modularity and flexibility, and in species with such rich communicative repertoires^17,23,41^. We can only hypothesize that interdependent causes such as social complexity, vocal redundance, and possible adaptive values of auditory YC might explain the presence of the vocalisation in the two species. In this context, auditory yawn contagion could indeed play a crucial role in intergroup communication and coordination within gelada multilevel societies, where different group units are often separated by medium to long distances^18^. During our analysis of the yawn responses in the playback experiments, we did not only consider the presence or absence of yawning but also the number of yawns produced. We discovered that yawn sounds produced by male members of the same group (i.e., more socially valuable individuals) elicited chains of more yawns in the responder after its first yawn, despite the probability of yawn response being similar when exposed to ingroup or outgroup yawn sounds. Importantly, here the interaction Condition*Group in GLMM_number of yawns_ showed that this significant effect was only present when exposed to yawn stimuli and not grunts. Indeed, if this result was due to autocorrelation of yawns (i.e., increased probability of a second yawn after a first yawn), we would expect a similar trend independently from the group membership of the yawn stimulus once the first yawn has been evoked in the tested subject. Our findings are consistent with previous studies that have demonstrated a social modulation in the contagiousness of observed (^3,4^, but see also^40^) or even just heard yawns (humans^16^, domestic dogs^9^). Notably, in those studies, yawn contagion was stronger among socially close group mates^3,9,16^ or individuals living together versus those unfamiliar with each other^4^. In our experiment, all the subjects were acoustically familiar with each other, as both groups could see and hear each other but could not interact (see Methods). This familiarity led to equally familiar yawn sounds (eliminating any neophobic effect) produced by individuals with different social values (ingroup vs. outgroup). The stronger contagious response observed towards ingroup male yawn sounds could potentially trigger a domino effect, increasing the probability of other group members perceiving and responding to previous yawns. In conclusion, our data present new insights: first, the importance of studying how the spread of contagious yawns among group members might lead to synchronizing group activities, and second, the possibility that, in such unique species, the acoustic component of yawns may carry information about the yawner's identity, allowing conspecifics to recognize the yawner (in parallel to what might occurs for domestic dogs with humans^9,42^). This study adds an important contribution to the research on the evolution of mimicry and behavioural contagion in the primate lineage, prompting further exploration on the role of sensory modalities beyond visual perception in these phenomena.

### Supplementary Information

Supplementary Information 1.

Supplementary Figure 1.

Supplementary Information 3.

Supplementary Table 1.

## Data Availability

The authors declare that all relevant data supporting the findings of this study have been submitted as Supplementary Information files.
