# Supplementary material for: The sound of yawns makes geladas yawn
Source: Sci Rep. 2024 Jan 7;14:361. doi: 10.1038/s41598-023-49797-5 (PMC10772098; doi:10.1038/s41598-023-49797-5)
Supplement: Supplementary file 2 — Supplementary Figure 1. [file 41598_2023_49797_MOESM2_ESM.docx]

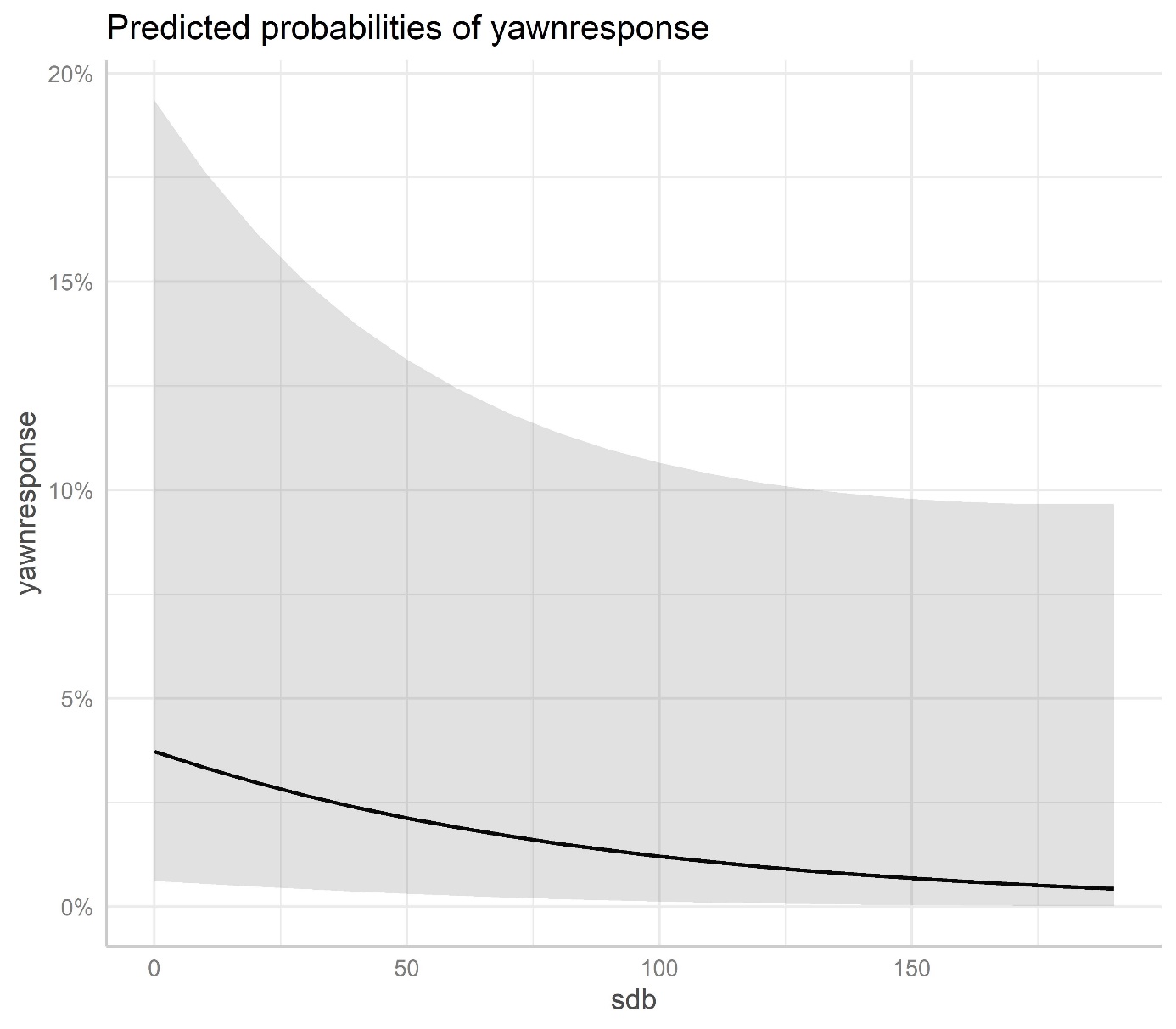


**Figure S1.** Effect plot showing the non-significant effect of time spent in self-directed behaviours on the likelihood of yawn response (GLMM_yawn response_, X^2^ = 2.55, p = 0.11).
