# Supplementary material for: The sound of yawns makes geladas yawn
Source: Sci Rep. 2024 Jan 7;14:361. doi: 10.1038/s41598-023-49797-5 (PMC10772098; doi:10.1038/s41598-023-49797-5)
Supplement: Supplementary file 4 — Supplementary Table 1. [file 41598_2023_49797_MOESM4_ESM.docx]

**Table S1.** Compositions of the different group units at the time of data collection (March 2023).

| **Group** | **Group unit (AMU/OMU)** | **Leader male ID** | **N° of adult females (>6 years)** | **N° of adult males (>6 years)** | **N° of subadult/juvenile males (3<years old<6)** | **N° of subadult/juvenile females (3<years old<6)** | **N° of infants**  **(<3years old)** |
| --- | --- | --- | --- | --- | --- | --- | --- |
| G1 | OMU 1 | Sumo | 20 | 0 | 3 | 4 | 2 |
| G1 | OMU 2 | Enzo | 5 | 0 | 2 | 4 | 6 |
| G1 | AMU | Rocco | 0 | 8 | 0 | 0 | 0 |
| G2 | OMU 3 | Biondo | 12 | 1 | 6 | 7 | 4 |
| G2 | OMU 4 | Giangi | 6 | 0 | 2 | 5 | 1 |
